# *N*,*N*-Bis(diphenyl­thio­phosphino­yl)-4-ethyl­aniline

**DOI:** 10.1107/S1600536812048416

**Published:** 2012-11-30

**Authors:** Peter A. Ajibade, Damian C. Onwudiwe, Bernard Omondi

**Affiliations:** a Department of Chemistry, University of Fort Hare, Private Bag X1314, Alice 5700, South Africa; b School of Chemistry and Physics, University of KwaZulu-Natal, Westville Campus, Private Bag X54001, Durban 4000, South Africa

## Abstract

The title compound, C_32_H_29_NP_2_S_2_, has two mol­ecules in the asymmetric unit, with an r.m.s. difference of 0.218 Å in their best-fit overlay. Both mol­ecules have a slightly distorted trigonal–planar N atom, bonded to two P^V^ atoms and a C atom of the 4-ethyl­phenyl unit. The P—N—P angles of 126.34 (11) and 125.98 (11)° are larger than the four C—N—P bond angles. The two S atoms are *trans* to one another with respect to the P—N—P angle. The crystal structure features C—H⋯π inter­actions. The methyl group in one of the mol­ecules is disordered over two sets of sites, with occupancies of 0.518 (6) and 0.482 (6).

## Related literature

For background to the chemistry of coordination compounds containing P—N bonds and for their applications, see: Hartley (1990 ▶); Greenwood & Earnshaw (1984 ▶); Balakrishna *et al.* (2000 ▶). For the ability of bis­(diphenyl­phosphino)alkyl­aniline derivatives to form chelates with transition metal ions, see: Biricik *et al.* (2007) ▶; Fei & Dyson (2005 ▶). For the synthesis of the title and related compounds, see: Fernández *et al.* (2005 ▶); Gaw *et al.* (2002 ▶); Fei *et al.* (2004 ▶); Slawin *et al.* (2005 ▶).
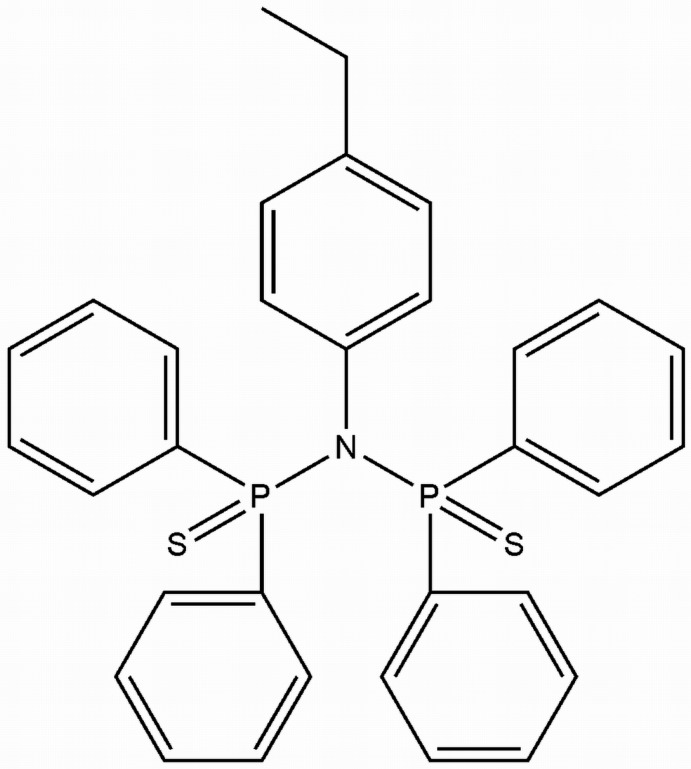


## Experimental

### 

#### Crystal data

C_32_H_29_NP_2_S_2_
*M* *_r_* = 553.62
Triclinic, 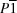

*a* = 9.6305 (11) Å
*b* = 13.8027 (14) Å
*c* = 21.360 (2) Å
α = 82.484 (2)°
β = 84.635 (2)°
γ = 79.975 (2)°
*V* = 2764.7 (5) Å^3^
*Z* = 4
Mo *K*α radiation
μ = 0.33 mm^−1^
*T* = 173 K
0.17 × 0.08 × 0.08 mm

#### Data collection

Bruker X8 APEXII 4K KappaCCD diffractometer
Absorption correction: multi-scan (*SADABS*; Bruker, 2008 ▶) *T*_min_ = 0.946, *T*_max_ = 0.974
56725 measured reflections
13522 independent reflections
9271 reflections with *I* > 2σ(*I*)
*R*_int_ = 0.054

#### Refinement

*R*[*F* ^2^ > 2σ(*F* ^2^)] = 0.042
*wR*(*F* ^2^) = 0.107
*S* = 1.00
13522 reflections
684 parameters
9 restraints
H-atom parameters constrained
Δρ_max_ = 0.82 e Å^−3^
Δρ_min_ = −0.36 e Å^−3^

### 

Data collection: *APEX2* (Bruker, 2008 ▶); cell refinement: *SAINT-Plus* (Bruker, 2008 ▶); data reduction: *SAINT-Plus* and *XPREP* (Bruker, 2008 ▶); program(s) used to solve structure: *SHELXS97* (Sheldrick, 2008 ▶); program(s) used to refine structure: *SHELXL97* (Sheldrick, 2008 ▶); molecular graphics: *ORTEP-3* (Farrugia, 2012 ▶); software used to prepare material for publication: *WinGX* (Farrugia, 2012 ▶).

## Supplementary Material

Crystal structure: contains datablock(s) global, I. DOI: 10.1107/S1600536812048416/fj2607sup1.cif

Structure factors: contains datablock(s) I. DOI: 10.1107/S1600536812048416/fj2607Isup2.hkl

Supplementary material file. DOI: 10.1107/S1600536812048416/fj2607Isup3.cml

Additional supplementary materials:  crystallographic information; 3D view; checkCIF report

## Figures and Tables

**Table 1:** Hydrogen-bond geometry (Å, °) *Cg*1 and *Cg*2 are the centroids of the C19–C24 and C57–C62 rings, respectively.

*D*—H⋯*A*	*D*—H	H⋯*A*	*D*⋯*A*	*D*—H⋯*A*
C17—H17⋯*Cg*2^i^	0.95	2.97	3.759 (3)	141
C64—H64*A*⋯*Cg*1^ii^	0.98	2.88	3.767 (3)	151

Symmetry codes: (i) 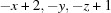; (ii) 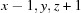.

**Fig. 1.**
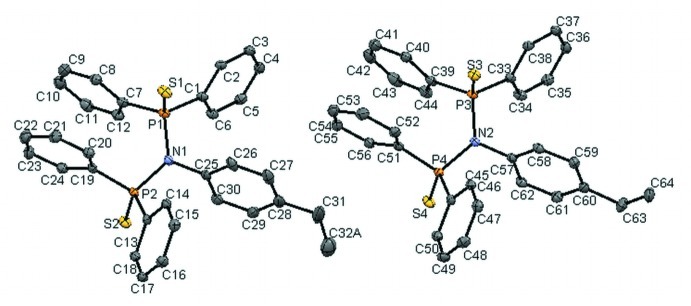
The asymmetric unit of the title compound with displacement ellipsoids drawn at the 50% probability level.

**Fig. 2.**
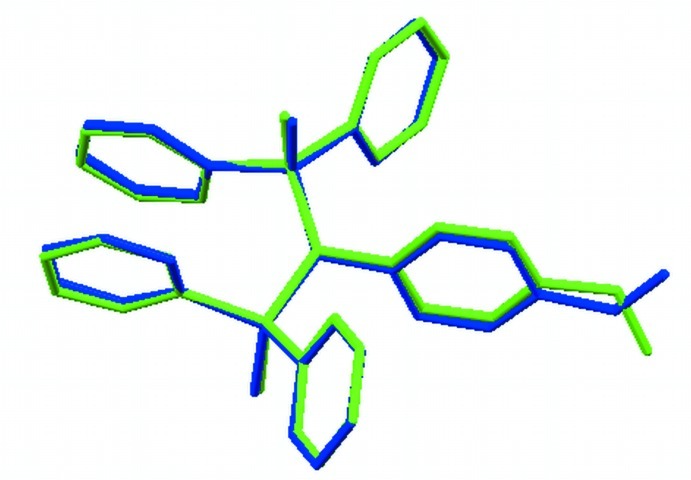
Overlay structure of the two molecules in the asymmetric unit.

## supplementary crystallographic information

### Comment

Compounds containing P—N bonds have been known for many years and their chemistry has been of considerable interest because of their applications in increasingly diverse fields (Hartley, 1990; Greenwood & Earnshaw, 1984) and in homogeneous catalysis as these ligands can be used for fine tuning the activity and selectivity of the catalyst (Balakrishna *et al.*, 2000). Bis(diphenylphosphino)alkylaniline derivatives are of particular interest among amino phosphines due to their stability and ability to form chelates with transition metal ions (Biricik *et al.*, 2007; Fei & Dyson, 2005). Synthesis of bis(diphenylphosphino)alkylaniline is through aminolysis of aniline (Fernández *et al.*, 2005; Gaw *et al.*, 2002) and incorporation of additional functional groups (Fei *et al.*, 2004; Slawin *et al.*, 2005).

The structure of (I) Fig. 1, has a slightly distorted trigonal planar N atom bound to two P^V^ atoms and a C atom of the *p*-ethylphenyl moiety in which the P–N–P angle is larger, 126.01 (11) and 126.28 (11)°, than the four C–N–P bond angles in the two molecules (between 116.03(13 and 117.10 (13) Å). The two S atoms are *trans* to one another with respect to the trigonal plane. The P–N bond distances 1.7162 (18), 1.7192 (18), 1.7134 (17) and 1.7212 (18) Å and P–S bond distances 1.9469 (8), 1.9468 (8), 1.9446 (8) and 1.9449 (8) Å are similar in both molecules and compare well to those of similar molecules in literature (Slawin *et al.*, 2005). The r.m.s. value of an overlay of the two molecules is 0.218 Å and shows major differences only between the ethyl moieties of the two molecules. The crystal lattice is stabilized by two C—H···π intermolecular interactions with *Cg*···H distances of 3.759 (3) and 3.767 (3) Å.

### Experimental

*N*,*N*-Bis(diphenylphosphino)ethylaniline is prepared by the aminolysis reaction of H_2_NC_6_H_4_—C_2_H_5_(4-C_2_H_5_) with two equivalents of Ph_2_PCl according to the procedure given in the literature (Hartley, 1990). Solid *N*,*N*-bis(diphenylphosphino)ethylaniline (0.20 g, 0.420 mmol) and S8 (0.027 g, 0.840 mmol) were dissolved in 20 ml of tetrahydrofuran and refluxed for 6 h. The volume was concentrated *in vacuo* to about 2 ml follow by the addition of 20 ml n-hexane to give [(Ph_2_P(S)_2_N—C_6_H_4_(C_2_H_5_)] as a white solid. Recrystallization in chloroform hexane gave crystals suitable for X-ray analysis.

### Crystal data

**Table:**

C_32_H_29_NP_2_S_2_	*Z* = 4
*M**_r_* = 553.62	*F*(000) = 1160
Triclinic, *P*1	*D*_x_ = 1.33 Mg m^−^^3^
Hall symbol: -P 1	Mo *K*α radiation, λ = 0.71073 Å
*a* = 9.6305 (11) Å	Cell parameters from 56725 reflections
*b* = 13.8027 (14) Å	θ = 1.0–28.2°
*c* = 21.360 (2) Å	µ = 0.33 mm^−^^1^
α = 82.484 (2)°	*T* = 173 K
β = 84.635 (2)°	Needle, colourless
γ = 79.975 (2)°	0.17 × 0.08 × 0.08 mm
*V* = 2764.7 (5) Å^3^	

### Data collection

**Table:**

Bruker X8 APEXII 4K KappaCCD diffractometer	9271 reflections with *I* > 2σ(*I*)
Graphite monochromator	*R*_int_ = 0.054
φ and ω scans	θ_max_ = 28.2°, θ_min_ = 1.0°
Absorption correction: multi-scan (*SADABS*; Bruker, 2008)	*h* = −12→12
*T*_min_ = 0.946, *T*_max_ = 0.974	*k* = −18→18
56725 measured reflections	*l* = −28→28
13522 independent reflections	

### Refinement

**Table:**

Refinement on *F*^2^	Primary atom site location: structure-invariant direct methods
Least-squares matrix: full	Secondary atom site location: difference Fourier map
*R*[*F*^2^ > 2σ(*F*^2^)] = 0.042	Hydrogen site location: inferred from neighbouring sites
*wR*(*F*^2^) = 0.107	H-atom parameters constrained
*S* = 1.00	*w* = 1/[σ^2^(*F*_o_^2^) + (0.0442*P*)^2^ + 1.5667*P*] where *P* = (*F*_o_^2^ + 2*F*_c_^2^)/3
13522 reflections	(Δ/σ)_max_ = 0.001
684 parameters	Δρ_max_ = 0.82 e Å^−^^3^
9 restraints	Δρ_min_ = −0.36 e Å^−^^3^

### Special details

**Table:**

Experimental. Carbon-bound H-atoms were placed in calculated positions [C—H = 0.98 Å for Me H atoms, 0.99 Å for Methylene H atoms and 0.95 for aromatic H atoms; *U*_iso_(H) = 1.2*U*_eq_(C) (1.5 for Me groups)] and were included in the refinement in the riding model approximation. Disorder: Disorder was found for one ethyl group in the *p*-ethylpyridyl moiety, which is not an uncommon situation. The disorder was modelled for C– and H– atoms using distance restraints and constraints, and PART instructions and the total occupancy was kept as 1 during the refinement. The carbon atom involved in disorder were modelled with anisotropic thermal parameters. The occupancy of the C atoms in the disordered ethyl moiety was fixed at 50:50 during the refinement.
Geometry. All e.s.d.'s (except the e.s.d. in the dihedral angle between two l.s. planes) are estimated using the full covariance matrix. The cell e.s.d.'s are taken into account individually in the estimation of e.s.d.'s in distances, angles and torsion angles; correlations between e.s.d.'s in cell parameters are only used when they are defined by crystal symmetry. An approximate (isotropic) treatment of cell e.s.d.'s is used for estimating e.s.d.'s involving l.s. planes.
Refinement. Refinement of *F*^2^ against ALL reflections. The weighted *R*-factor *wR* and goodness of fit *S* are based on *F*^2^, conventional *R*-factors *R* are based on *F*, with *F* set to zero for negative *F*^2^. The threshold expression of *F*^2^ > σ(*F*^2^) is used only for calculating *R*-factors(gt) *etc*. and is not relevant to the choice of reflections for refinement. *R*-factors based on *F*^2^ are statistically about twice as large as those based on *F*, and *R*- factors based on ALL data will be even larger.

### Fractional atomic coordinates and isotropic or equivalent isotropic displacement parameters (Å^2^)

**Table:**

	*x*	*y*	*z*	*U*_iso_*/*U*_eq_	Occ. (<1)
C1	0.5587 (2)	0.33146 (16)	0.20639 (10)	0.0147 (4)	
C2	0.4212 (2)	0.31222 (16)	0.22027 (10)	0.0158 (4)	
H2	0.389	0.2643	0.1996	0.019*	
C3	0.3308 (2)	0.36277 (17)	0.26418 (11)	0.0180 (5)	
H3	0.2371	0.3493	0.2733	0.022*	
C4	0.3767 (2)	0.43249 (17)	0.29454 (11)	0.0184 (5)	
H4	0.3149	0.4666	0.3247	0.022*	
C5	0.5134 (2)	0.45267 (16)	0.28089 (11)	0.0178 (5)	
H5	0.5449	0.5007	0.3018	0.021*	
C6	0.6041 (2)	0.40305 (16)	0.23693 (11)	0.0170 (5)	
H6	0.6972	0.4176	0.2275	0.02*	
C7	0.6830 (2)	0.35878 (16)	0.07850 (11)	0.0163 (4)	
C8	0.6379 (2)	0.34382 (18)	0.02078 (11)	0.0202 (5)	
H8	0.603	0.2848	0.0169	0.024*	
C9	0.6443 (2)	0.4158 (2)	−0.03108 (12)	0.0258 (5)	
H9	0.6149	0.4051	−0.0705	0.031*	
C10	0.6929 (2)	0.50268 (19)	−0.02589 (12)	0.0269 (6)	
H10	0.6965	0.5515	−0.0615	0.032*	
C11	0.7366 (2)	0.51829 (18)	0.03159 (12)	0.0241 (5)	
H11	0.7694	0.5782	0.0354	0.029*	
C12	0.7326 (2)	0.44653 (17)	0.08364 (11)	0.0198 (5)	
H12	0.7637	0.4572	0.1227	0.024*	
C13	1.0769 (2)	0.10323 (16)	0.16047 (10)	0.0138 (4)	
C14	0.9979 (2)	0.02726 (16)	0.17924 (10)	0.0152 (4)	
H14	0.8976	0.0411	0.1801	0.018*	
C15	1.0657 (2)	−0.06832 (17)	0.19667 (11)	0.0176 (5)	
H15	1.0118	−0.1195	0.2108	0.021*	
C16	1.2126 (2)	−0.08925 (17)	0.19343 (11)	0.0199 (5)	
H16	1.2589	−0.155	0.2047	0.024*	
C17	1.2917 (2)	−0.01454 (18)	0.17376 (11)	0.0193 (5)	
H17	1.3921	−0.0293	0.1712	0.023*	
C18	1.2246 (2)	0.08165 (17)	0.15790 (10)	0.0160 (4)	
H18	1.2789	0.133	0.1452	0.019*	
C19	0.9629 (2)	0.22248 (17)	0.05160 (10)	0.0151 (4)	
C20	0.9154 (2)	0.14218 (17)	0.03165 (11)	0.0172 (5)	
H20	0.8917	0.0895	0.0618	0.021*	
C21	0.9029 (2)	0.13954 (18)	−0.03239 (11)	0.0212 (5)	
H21	0.8696	0.0855	−0.046	0.025*	
C22	0.9392 (2)	0.21577 (19)	−0.07646 (12)	0.0253 (5)	
H22	0.93	0.2139	−0.1202	0.03*	
C23	0.9888 (2)	0.29476 (19)	−0.05695 (11)	0.0233 (5)	
H23	1.0149	0.3463	−0.0874	0.028*	
C24	1.0002 (2)	0.29855 (17)	0.00685 (11)	0.0178 (5)	
H24	1.0335	0.3529	0.0201	0.021*	
C25	0.8366 (2)	0.21872 (17)	0.24500 (10)	0.0159 (4)	
C26	0.7654 (2)	0.14545 (17)	0.27729 (11)	0.0212 (5)	
H26	0.7177	0.1083	0.2544	0.025*	
C27	0.7638 (3)	0.12657 (19)	0.34268 (12)	0.0271 (6)	
H27	0.7131	0.0773	0.3641	0.033*	
C28	0.8352 (2)	0.1784 (2)	0.37793 (12)	0.0280 (6)	
C29	0.9056 (2)	0.2508 (2)	0.34481 (12)	0.0268 (6)	
H29	0.9549	0.2871	0.3676	0.032*	
C30	0.9065 (2)	0.27200 (18)	0.27942 (11)	0.0201 (5)	
H30	0.9548	0.3227	0.2582	0.024*	
C31A	0.8203 (5)	0.1365 (5)	0.4457 (3)	0.0251 (14)	0.482 (6)
H31A	0.8463	0.0634	0.4503	0.03*	0.482 (6)
H31B	0.722	0.1542	0.4637	0.03*	0.482 (6)
C32A	0.9219 (6)	0.1829 (4)	0.4788 (3)	0.0328 (16)	0.482 (6)
H32A	1.0175	0.1681	0.4585	0.049*	0.482 (6)
H32B	0.9217	0.1555	0.5235	0.049*	0.482 (6)
H32C	0.8916	0.2548	0.4756	0.049*	0.482 (6)
C32B	0.9574 (6)	0.0888 (6)	0.4745 (3)	0.072 (3)	0.518 (6)
H32D	0.9476	0.0255	0.4606	0.108*	0.518 (6)
H32E	0.9522	0.0824	0.5209	0.108*	0.518 (6)
H32F	1.0487	0.1068	0.4576	0.108*	0.518 (6)
C31B	0.8439 (6)	0.1653 (4)	0.4514 (3)	0.0204 (15)*	0.518 (6)
H31C	0.8568	0.2292	0.4646	0.025*	0.518 (6)
H31D	0.7528	0.1493	0.4719	0.025*	0.518 (6)
C33	0.0832 (2)	0.32778 (16)	0.71094 (10)	0.0136 (4)	
C34	0.1283 (2)	0.39978 (16)	0.74106 (11)	0.0180 (5)	
H34	0.2223	0.4129	0.7325	0.022*	
C35	0.0356 (2)	0.45200 (17)	0.78348 (11)	0.0182 (5)	
H35	0.0668	0.5003	0.8042	0.022*	
C36	−0.1018 (2)	0.43402 (17)	0.79578 (11)	0.0181 (5)	
H36	−0.1646	0.4698	0.8249	0.022*	
C37	−0.1477 (2)	0.36362 (17)	0.76552 (11)	0.0179 (5)	
H37	−0.2424	0.3517	0.7736	0.022*	
C38	−0.0557 (2)	0.31046 (16)	0.72330 (10)	0.0148 (4)	
H38	−0.0875	0.2622	0.7028	0.018*	
C39	0.1969 (2)	0.35422 (16)	0.58254 (11)	0.0162 (5)	
C40	0.1467 (2)	0.33689 (18)	0.52653 (11)	0.0197 (5)	
H40	0.1147	0.2762	0.5242	0.024*	
C41	0.1434 (2)	0.4083 (2)	0.47428 (12)	0.0269 (6)	
H41	0.1103	0.3959	0.436	0.032*	
C42	0.1880 (2)	0.4973 (2)	0.47764 (13)	0.0303 (6)	
H42	0.1853	0.546	0.4417	0.036*	
C43	0.2364 (2)	0.51555 (18)	0.53309 (13)	0.0274 (6)	
H43	0.2665	0.5769	0.5353	0.033*	
C44	0.2412 (2)	0.44471 (17)	0.58575 (12)	0.0215 (5)	
H44	0.2745	0.4576	0.6238	0.026*	
C45	0.6032 (2)	0.10402 (16)	0.65769 (10)	0.0141 (4)	
C46	0.5259 (2)	0.02773 (16)	0.67787 (10)	0.0150 (4)	
H46	0.4255	0.0414	0.6813	0.018*	
C47	0.5958 (2)	−0.06828 (17)	0.69293 (11)	0.0180 (5)	
H47	0.5433	−0.1202	0.7074	0.022*	
C48	0.7427 (2)	−0.08827 (17)	0.68685 (11)	0.0209 (5)	
H48	0.7903	−0.154	0.697	0.025*	
C49	0.8201 (2)	−0.01322 (17)	0.66622 (11)	0.0203 (5)	
H49	0.9204	−0.0275	0.6618	0.024*	
C50	0.7507 (2)	0.08337 (17)	0.65195 (11)	0.0172 (5)	
H50	0.8038	0.1352	0.6383	0.021*	
C51	0.4775 (2)	0.22518 (16)	0.55163 (10)	0.0145 (4)	
C52	0.4342 (2)	0.14306 (17)	0.53204 (11)	0.0172 (5)	
H52	0.4193	0.0879	0.5619	0.021*	
C53	0.4132 (2)	0.14256 (18)	0.46862 (11)	0.0218 (5)	
H53	0.3824	0.0873	0.4553	0.026*	
C54	0.4369 (2)	0.22218 (19)	0.42477 (12)	0.0245 (5)	
H54	0.422	0.2214	0.3815	0.029*	
C55	0.4823 (2)	0.30330 (19)	0.44380 (11)	0.0225 (5)	
H55	0.4996	0.3575	0.4135	0.027*	
C56	0.5024 (2)	0.30489 (17)	0.50684 (11)	0.0181 (5)	
H56	0.5333	0.3604	0.5198	0.022*	
C57	0.3692 (2)	0.21982 (16)	0.74692 (10)	0.0137 (4)	
C58	0.3021 (2)	0.14614 (16)	0.78082 (11)	0.0164 (4)	
H58	0.2532	0.108	0.7592	0.02*	
C59	0.3063 (2)	0.12780 (17)	0.84639 (11)	0.0172 (5)	
H59	0.258	0.0782	0.8691	0.021*	
C60	0.3801 (2)	0.18089 (17)	0.87935 (11)	0.0182 (5)	
C61	0.4467 (2)	0.25418 (17)	0.84421 (11)	0.0197 (5)	
H61	0.4977	0.2914	0.8655	0.024*	
C62	0.4413 (2)	0.27475 (17)	0.77930 (11)	0.0171 (5)	
H62	0.4865	0.3261	0.7568	0.021*	
C63	0.3924 (3)	0.16013 (19)	0.95017 (11)	0.0247 (5)	
H63A	0.3512	0.2212	0.9694	0.03*	
H63B	0.4941	0.1456	0.958	0.03*	
C64	0.3223 (3)	0.0759 (2)	0.98406 (12)	0.0286 (6)	
H64A	0.2205	0.0907	0.9788	0.043*	
H64B	0.3382	0.0681	1.0292	0.043*	
H64C	0.3628	0.0145	0.9661	0.043*	
N1	0.83303 (17)	0.24265 (13)	0.17644 (8)	0.0141 (4)	
N2	0.35942 (17)	0.24277 (13)	0.67843 (8)	0.0128 (4)	
P1	0.66983 (5)	0.26622 (4)	0.14637 (3)	0.01357 (12)	
P2	0.99377 (5)	0.22740 (4)	0.13353 (3)	0.01236 (12)	
P3	0.19389 (5)	0.26192 (4)	0.65115 (3)	0.01247 (12)	
P4	0.51696 (5)	0.22863 (4)	0.63272 (3)	0.01222 (12)	
S1	0.59676 (6)	0.15083 (4)	0.12805 (3)	0.01966 (13)	
S2	1.10868 (6)	0.32748 (4)	0.14140 (3)	0.01785 (12)	
S3	0.12578 (5)	0.14324 (4)	0.63558 (3)	0.01649 (12)	
S4	0.63275 (6)	0.32894 (4)	0.63852 (3)	0.01812 (12)	

### Atomic displacement parameters (Å^2^)

**Table:**

	*U*^11^	*U*^22^	*U*^33^	*U*^12^	*U*^13^	*U*^23^
C1	0.0126 (9)	0.0130 (11)	0.0178 (11)	0.0006 (8)	−0.0011 (8)	−0.0030 (9)
C2	0.0136 (9)	0.0149 (11)	0.0182 (11)	−0.0002 (8)	−0.0027 (8)	−0.0016 (9)
C3	0.0124 (9)	0.0201 (12)	0.0205 (12)	−0.0009 (8)	−0.0005 (8)	−0.0017 (9)
C4	0.0192 (10)	0.0180 (12)	0.0159 (11)	0.0037 (8)	−0.0011 (8)	−0.0034 (9)
C5	0.0210 (11)	0.0125 (11)	0.0197 (12)	0.0007 (8)	−0.0052 (9)	−0.0029 (9)
C6	0.0147 (10)	0.0152 (11)	0.0213 (12)	−0.0030 (8)	−0.0013 (8)	−0.0023 (9)
C7	0.0122 (9)	0.0148 (11)	0.0215 (12)	−0.0013 (8)	−0.0004 (8)	−0.0026 (9)
C8	0.0166 (10)	0.0221 (13)	0.0222 (12)	−0.0021 (9)	−0.0016 (9)	−0.0046 (10)
C9	0.0216 (11)	0.0340 (15)	0.0202 (12)	−0.0021 (10)	−0.0024 (9)	−0.0003 (11)
C10	0.0214 (11)	0.0270 (14)	0.0273 (14)	0.0002 (10)	0.0029 (10)	0.0066 (11)
C11	0.0214 (11)	0.0167 (12)	0.0330 (14)	−0.0052 (9)	0.0041 (10)	0.0006 (10)
C12	0.0172 (10)	0.0202 (12)	0.0230 (12)	−0.0051 (9)	−0.0011 (9)	−0.0036 (10)
C13	0.0138 (9)	0.0150 (11)	0.0126 (10)	−0.0007 (8)	−0.0004 (8)	−0.0039 (8)
C14	0.0125 (9)	0.0181 (12)	0.0150 (11)	−0.0016 (8)	0.0002 (8)	−0.0045 (9)
C15	0.0205 (11)	0.0151 (12)	0.0175 (11)	−0.0038 (8)	0.0003 (8)	−0.0030 (9)
C16	0.0207 (11)	0.0175 (12)	0.0196 (12)	0.0044 (9)	−0.0023 (9)	−0.0044 (9)
C17	0.0131 (10)	0.0256 (13)	0.0185 (12)	0.0024 (9)	−0.0022 (8)	−0.0071 (10)
C18	0.0128 (9)	0.0202 (12)	0.0159 (11)	−0.0024 (8)	−0.0002 (8)	−0.0059 (9)
C19	0.0117 (9)	0.0181 (12)	0.0150 (11)	−0.0006 (8)	0.0011 (8)	−0.0040 (9)
C20	0.0162 (10)	0.0160 (12)	0.0194 (12)	−0.0014 (8)	−0.0011 (8)	−0.0040 (9)
C21	0.0215 (11)	0.0218 (13)	0.0215 (12)	−0.0013 (9)	−0.0036 (9)	−0.0081 (10)
C22	0.0278 (12)	0.0303 (15)	0.0173 (12)	0.0001 (10)	−0.0025 (9)	−0.0069 (10)
C23	0.0241 (11)	0.0270 (14)	0.0172 (12)	−0.0048 (10)	0.0003 (9)	0.0027 (10)
C24	0.0161 (10)	0.0172 (12)	0.0203 (12)	−0.0041 (8)	−0.0005 (8)	−0.0022 (9)
C25	0.0136 (9)	0.0171 (11)	0.0143 (11)	0.0027 (8)	0.0018 (8)	−0.0013 (9)
C26	0.0204 (11)	0.0146 (12)	0.0249 (13)	0.0016 (9)	0.0076 (9)	−0.0014 (10)
C27	0.0259 (12)	0.0202 (13)	0.0268 (14)	0.0068 (10)	0.0097 (10)	0.0063 (11)
C28	0.0203 (11)	0.0355 (15)	0.0198 (13)	0.0110 (10)	0.0020 (9)	0.0050 (11)
C29	0.0208 (11)	0.0393 (16)	0.0181 (12)	0.0034 (10)	−0.0040 (9)	−0.0052 (11)
C30	0.0165 (10)	0.0236 (13)	0.0189 (12)	−0.0005 (9)	0.0010 (8)	−0.0025 (10)
C31A	0.020 (3)	0.038 (4)	0.022 (3)	−0.015 (3)	−0.005 (2)	−0.004 (3)
C32A	0.034 (3)	0.034 (4)	0.027 (3)	−0.001 (2)	−0.005 (2)	0.004 (2)
C32B	0.088 (6)	0.084 (6)	0.021 (3)	0.048 (5)	−0.006 (3)	−0.002 (3)
C33	0.0125 (9)	0.0105 (10)	0.0167 (11)	0.0010 (7)	−0.0007 (8)	−0.0015 (8)
C34	0.0144 (10)	0.0132 (11)	0.0270 (13)	−0.0024 (8)	−0.0010 (9)	−0.0047 (9)
C35	0.0198 (10)	0.0145 (11)	0.0205 (12)	−0.0012 (8)	−0.0032 (9)	−0.0042 (9)
C36	0.0186 (10)	0.0170 (12)	0.0166 (11)	0.0025 (8)	0.0009 (8)	−0.0030 (9)
C37	0.0123 (9)	0.0182 (12)	0.0221 (12)	−0.0005 (8)	−0.0001 (8)	−0.0015 (9)
C38	0.0130 (9)	0.0137 (11)	0.0176 (11)	−0.0010 (8)	−0.0022 (8)	−0.0024 (9)
C39	0.0101 (9)	0.0139 (11)	0.0235 (12)	−0.0007 (8)	0.0015 (8)	−0.0010 (9)
C40	0.0143 (10)	0.0211 (12)	0.0226 (12)	−0.0013 (8)	−0.0003 (8)	−0.0016 (10)
C41	0.0185 (11)	0.0339 (15)	0.0250 (13)	−0.0011 (10)	0.0009 (9)	0.0025 (11)
C42	0.0207 (12)	0.0289 (15)	0.0321 (15)	0.0039 (10)	0.0060 (10)	0.0142 (12)
C43	0.0208 (11)	0.0155 (12)	0.0423 (16)	−0.0027 (9)	0.0073 (10)	0.0034 (11)
C44	0.0174 (10)	0.0178 (12)	0.0284 (13)	−0.0028 (9)	0.0017 (9)	−0.0014 (10)
C45	0.0153 (10)	0.0147 (11)	0.0121 (10)	0.0002 (8)	−0.0002 (8)	−0.0049 (8)
C46	0.0132 (9)	0.0169 (11)	0.0156 (11)	−0.0009 (8)	−0.0013 (8)	−0.0064 (9)
C47	0.0207 (11)	0.0158 (12)	0.0177 (11)	−0.0026 (8)	−0.0005 (9)	−0.0042 (9)
C48	0.0232 (11)	0.0159 (12)	0.0225 (12)	0.0052 (9)	−0.0060 (9)	−0.0067 (10)
C49	0.0139 (10)	0.0221 (13)	0.0245 (12)	0.0029 (8)	−0.0025 (9)	−0.0083 (10)
C50	0.0140 (10)	0.0191 (12)	0.0192 (11)	−0.0029 (8)	−0.0009 (8)	−0.0046 (9)
C51	0.0110 (9)	0.0158 (11)	0.0160 (11)	−0.0003 (8)	0.0013 (8)	−0.0039 (9)
C52	0.0162 (10)	0.0163 (12)	0.0185 (12)	0.0018 (8)	−0.0017 (8)	−0.0051 (9)
C53	0.0214 (11)	0.0222 (13)	0.0227 (13)	0.0019 (9)	−0.0044 (9)	−0.0110 (10)
C54	0.0222 (11)	0.0331 (15)	0.0166 (12)	0.0031 (10)	−0.0028 (9)	−0.0063 (11)
C55	0.0191 (11)	0.0278 (14)	0.0179 (12)	−0.0007 (9)	0.0015 (9)	0.0014 (10)
C56	0.0141 (10)	0.0194 (12)	0.0196 (12)	−0.0017 (8)	0.0011 (8)	−0.0012 (9)
C57	0.0121 (9)	0.0132 (11)	0.0153 (11)	0.0001 (8)	−0.0001 (8)	−0.0032 (8)
C58	0.0144 (10)	0.0157 (11)	0.0199 (12)	−0.0020 (8)	−0.0003 (8)	−0.0055 (9)
C59	0.0169 (10)	0.0152 (11)	0.0184 (11)	−0.0021 (8)	0.0015 (8)	−0.0011 (9)
C60	0.0174 (10)	0.0194 (12)	0.0165 (11)	0.0019 (8)	−0.0002 (8)	−0.0040 (9)
C61	0.0196 (10)	0.0209 (12)	0.0204 (12)	−0.0051 (9)	−0.0012 (9)	−0.0073 (10)
C62	0.0157 (10)	0.0158 (11)	0.0208 (12)	−0.0053 (8)	0.0002 (8)	−0.0032 (9)
C63	0.0323 (13)	0.0279 (14)	0.0137 (11)	−0.0035 (10)	−0.0032 (9)	−0.0034 (10)
C64	0.0338 (14)	0.0337 (15)	0.0160 (12)	−0.0034 (11)	0.0017 (10)	−0.0001 (11)
N1	0.0125 (8)	0.0149 (9)	0.0145 (9)	−0.0013 (7)	0.0010 (7)	−0.0025 (7)
N2	0.0112 (8)	0.0132 (9)	0.0142 (9)	−0.0024 (6)	0.0009 (6)	−0.0034 (7)
P1	0.0114 (2)	0.0116 (3)	0.0184 (3)	−0.00273 (19)	0.0005 (2)	−0.0043 (2)
P2	0.0112 (2)	0.0123 (3)	0.0142 (3)	−0.00285 (19)	0.00019 (19)	−0.0034 (2)
P3	0.0108 (2)	0.0107 (3)	0.0163 (3)	−0.00226 (19)	−0.00034 (19)	−0.0027 (2)
P4	0.0110 (2)	0.0121 (3)	0.0142 (3)	−0.00277 (19)	0.00019 (19)	−0.0036 (2)
S1	0.0173 (3)	0.0155 (3)	0.0286 (3)	−0.0069 (2)	0.0029 (2)	−0.0094 (2)
S2	0.0171 (2)	0.0173 (3)	0.0216 (3)	−0.0079 (2)	0.0007 (2)	−0.0064 (2)
S3	0.0162 (2)	0.0135 (3)	0.0214 (3)	−0.0049 (2)	−0.0010 (2)	−0.0049 (2)
S4	0.0168 (2)	0.0176 (3)	0.0225 (3)	−0.0085 (2)	0.0021 (2)	−0.0066 (2)

### Geometric parameters (Å, º)

**Table:**

C1—C2	1.393 (3)	C33—C34	1.400 (3)
C1—C6	1.402 (3)	C33—P3	1.818 (2)
C1—P1	1.819 (2)	C34—C35	1.390 (3)
C2—C3	1.390 (3)	C34—H34	0.95
C2—H2	0.95	C35—C36	1.384 (3)
C3—C4	1.381 (3)	C35—H35	0.95
C3—H3	0.95	C36—C37	1.387 (3)
C4—C5	1.389 (3)	C36—H36	0.95
C4—H4	0.95	C37—C38	1.391 (3)
C5—C6	1.386 (3)	C37—H37	0.95
C5—H5	0.95	C38—H38	0.95
C6—H6	0.95	C39—C40	1.395 (3)
C7—C8	1.396 (3)	C39—C44	1.400 (3)
C7—C12	1.398 (3)	C39—P3	1.813 (2)
C7—P1	1.813 (2)	C40—C41	1.388 (3)
C8—C9	1.392 (3)	C40—H40	0.95
C8—H8	0.95	C41—C42	1.383 (4)
C9—C10	1.382 (4)	C41—H41	0.95
C9—H9	0.95	C42—C43	1.381 (4)
C10—C11	1.389 (4)	C42—H42	0.95
C10—H10	0.95	C43—C44	1.389 (3)
C11—C12	1.391 (3)	C43—H43	0.95
C11—H11	0.95	C44—H44	0.95
C12—H12	0.95	C45—C46	1.394 (3)
C13—C14	1.397 (3)	C45—C50	1.397 (3)
C13—C18	1.398 (3)	C45—P4	1.811 (2)
C13—P2	1.806 (2)	C46—C47	1.390 (3)
C14—C15	1.387 (3)	C46—H46	0.95
C14—H14	0.95	C47—C48	1.391 (3)
C15—C16	1.391 (3)	C47—H47	0.95
C15—H15	0.95	C48—C49	1.382 (3)
C16—C17	1.386 (3)	C48—H48	0.95
C16—H16	0.95	C49—C50	1.392 (3)
C17—C18	1.386 (3)	C49—H49	0.95
C17—H17	0.95	C50—H50	0.95
C18—H18	0.95	C51—C52	1.398 (3)
C19—C24	1.396 (3)	C51—C56	1.399 (3)
C19—C20	1.398 (3)	C51—P4	1.816 (2)
C19—P2	1.814 (2)	C52—C53	1.389 (3)
C20—C21	1.390 (3)	C52—H52	0.95
C20—H20	0.95	C53—C54	1.384 (4)
C21—C22	1.386 (3)	C53—H53	0.95
C21—H21	0.95	C54—C55	1.391 (4)
C22—C23	1.388 (4)	C54—H54	0.95
C22—H22	0.95	C55—C56	1.382 (3)
C23—C24	1.386 (3)	C55—H55	0.95
C23—H23	0.95	C56—H56	0.95
C24—H24	0.95	C57—C58	1.387 (3)
C25—C26	1.392 (3)	C57—C62	1.395 (3)
C25—C30	1.394 (3)	C57—N2	1.464 (3)
C25—N1	1.461 (3)	C58—C59	1.393 (3)
C26—C27	1.386 (3)	C58—H58	0.95
C26—H26	0.95	C59—C60	1.396 (3)
C27—C28	1.400 (4)	C59—H59	0.95
C27—H27	0.95	C60—C61	1.391 (3)
C28—C29	1.385 (4)	C60—C63	1.514 (3)
C28—C31A	1.491 (6)	C61—C62	1.382 (3)
C28—C31B	1.565 (7)	C61—H61	0.95
C29—C30	1.389 (3)	C62—H62	0.95
C29—H29	0.95	C63—C64	1.514 (4)
C30—H30	0.95	C63—H63A	0.99
C31A—C32A	1.527 (2)	C63—H63B	0.99
C31A—H31A	0.99	C64—H64A	0.98
C31A—H31B	0.99	C64—H64B	0.98
C32A—H32A	0.98	C64—H64C	0.98
C32A—H32B	0.98	N1—P1	1.7160 (17)
C32A—H32C	0.98	N1—P2	1.7183 (18)
C32B—C31B	1.455 (8)	N2—P3	1.7138 (17)
C32B—H32D	0.98	N2—P4	1.7214 (17)
C32B—H32E	0.98	P1—S1	1.9467 (8)
C32B—H32F	0.98	P2—S2	1.9466 (8)
C31B—H31C	0.99	P3—S3	1.9446 (8)
C31B—H31D	0.99	P4—S4	1.9451 (8)
C33—C38	1.394 (3)		
			
C2—C1—C6	119.05 (19)	C37—C36—H36	120.1
C2—C1—P1	118.31 (17)	C36—C37—C38	120.2 (2)
C6—C1—P1	122.58 (16)	C36—C37—H37	119.9
C3—C2—C1	120.4 (2)	C38—C37—H37	119.9
C3—C2—H2	119.8	C37—C38—C33	120.3 (2)
C1—C2—H2	119.8	C37—C38—H38	119.9
C4—C3—C2	120.2 (2)	C33—C38—H38	119.9
C4—C3—H3	119.9	C40—C39—C44	119.4 (2)
C2—C3—H3	119.9	C40—C39—P3	119.23 (18)
C3—C4—C5	120.0 (2)	C44—C39—P3	121.27 (18)
C3—C4—H4	120	C41—C40—C39	120.0 (2)
C5—C4—H4	120	C41—C40—H40	120
C6—C5—C4	120.3 (2)	C39—C40—H40	120
C6—C5—H5	119.9	C42—C41—C40	120.3 (2)
C4—C5—H5	119.9	C42—C41—H41	119.8
C5—C6—C1	120.1 (2)	C40—C41—H41	119.8
C5—C6—H6	119.9	C43—C42—C41	120.1 (2)
C1—C6—H6	119.9	C43—C42—H42	120
C8—C7—C12	119.5 (2)	C41—C42—H42	120
C8—C7—P1	119.45 (18)	C42—C43—C44	120.4 (2)
C12—C7—P1	121.05 (17)	C42—C43—H43	119.8
C9—C8—C7	119.7 (2)	C44—C43—H43	119.8
C9—C8—H8	120.1	C43—C44—C39	119.8 (2)
C7—C8—H8	120.1	C43—C44—H44	120.1
C10—C9—C8	120.8 (2)	C39—C44—H44	120.1
C10—C9—H9	119.6	C46—C45—C50	119.8 (2)
C8—C9—H9	119.6	C46—C45—P4	121.54 (15)
C9—C10—C11	119.7 (2)	C50—C45—P4	118.51 (17)
C9—C10—H10	120.2	C47—C46—C45	119.97 (19)
C11—C10—H10	120.2	C47—C46—H46	120
C10—C11—C12	120.2 (2)	C45—C46—H46	120
C10—C11—H11	119.9	C46—C47—C48	119.9 (2)
C12—C11—H11	119.9	C46—C47—H47	120.1
C11—C12—C7	120.1 (2)	C48—C47—H47	120.1
C11—C12—H12	119.9	C49—C48—C47	120.5 (2)
C7—C12—H12	119.9	C49—C48—H48	119.8
C14—C13—C18	119.6 (2)	C47—C48—H48	119.8
C14—C13—P2	121.53 (15)	C48—C49—C50	119.9 (2)
C18—C13—P2	118.75 (17)	C48—C49—H49	120.1
C15—C14—C13	120.00 (19)	C50—C49—H49	120.1
C15—C14—H14	120	C49—C50—C45	120.0 (2)
C13—C14—H14	120	C49—C50—H50	120
C14—C15—C16	120.0 (2)	C45—C50—H50	120
C14—C15—H15	120	C52—C51—C56	119.5 (2)
C16—C15—H15	120	C52—C51—P4	122.32 (17)
C17—C16—C15	120.2 (2)	C56—C51—P4	117.95 (17)
C17—C16—H16	119.9	C53—C52—C51	119.7 (2)
C15—C16—H16	119.9	C53—C52—H52	120.2
C16—C17—C18	120.0 (2)	C51—C52—H52	120.2
C16—C17—H17	120	C54—C53—C52	120.3 (2)
C18—C17—H17	120	C54—C53—H53	119.8
C17—C18—C13	120.1 (2)	C52—C53—H53	119.8
C17—C18—H18	119.9	C53—C54—C55	120.2 (2)
C13—C18—H18	119.9	C53—C54—H54	119.9
C24—C19—C20	119.7 (2)	C55—C54—H54	119.9
C24—C19—P2	117.55 (17)	C56—C55—C54	119.9 (2)
C20—C19—P2	122.52 (17)	C56—C55—H55	120.1
C21—C20—C19	119.9 (2)	C54—C55—H55	120.1
C21—C20—H20	120.1	C55—C56—C51	120.3 (2)
C19—C20—H20	120.1	C55—C56—H56	119.8
C22—C21—C20	120.1 (2)	C51—C56—H56	119.8
C22—C21—H21	120	C58—C57—C62	119.1 (2)
C20—C21—H21	120	C58—C57—N2	120.59 (19)
C21—C22—C23	120.2 (2)	C62—C57—N2	120.23 (19)
C21—C22—H22	119.9	C57—C58—C59	120.3 (2)
C23—C22—H22	119.9	C57—C58—H58	119.8
C24—C23—C22	120.1 (2)	C59—C58—H58	119.8
C24—C23—H23	119.9	C58—C59—C60	121.3 (2)
C22—C23—H23	119.9	C58—C59—H59	119.4
C23—C24—C19	120.0 (2)	C60—C59—H59	119.4
C23—C24—H24	120	C61—C60—C59	117.3 (2)
C19—C24—H24	120	C61—C60—C63	119.9 (2)
C26—C25—C30	119.0 (2)	C59—C60—C63	122.9 (2)
C26—C25—N1	120.7 (2)	C62—C61—C60	122.2 (2)
C30—C25—N1	120.3 (2)	C62—C61—H61	118.9
C27—C26—C25	120.3 (2)	C60—C61—H61	118.9
C27—C26—H26	119.9	C61—C62—C57	119.8 (2)
C25—C26—H26	119.9	C61—C62—H62	120.1
C26—C27—C28	121.5 (2)	C57—C62—H62	120.1
C26—C27—H27	119.2	C60—C63—C64	116.2 (2)
C28—C27—H27	119.2	C60—C63—H63A	108.2
C29—C28—C27	117.2 (2)	C64—C63—H63A	108.2
C29—C28—C31A	134.5 (3)	C60—C63—H63B	108.2
C27—C28—C31A	108.2 (3)	C64—C63—H63B	108.2
C29—C28—C31B	115.3 (3)	H63A—C63—H63B	107.4
C27—C28—C31B	127.5 (3)	C63—C64—H64A	109.5
C28—C29—C30	122.2 (2)	C63—C64—H64B	109.5
C28—C29—H29	118.9	H64A—C64—H64B	109.5
C30—C29—H29	118.9	C63—C64—H64C	109.5
C29—C30—C25	119.8 (2)	H64A—C64—H64C	109.5
C29—C30—H30	120.1	H64B—C64—H64C	109.5
C25—C30—H30	120.1	C25—N1—P1	116.96 (13)
C28—C31A—C32A	105.0 (4)	C25—N1—P2	116.05 (13)
C28—C31A—H31A	110.8	P1—N1—P2	126.34 (11)
C32A—C31A—H31A	110.8	C57—N2—P3	117.07 (13)
C28—C31A—H31B	110.8	C57—N2—P4	116.11 (13)
C32A—C31A—H31B	110.8	P3—N2—P4	125.98 (11)
H31A—C31A—H31B	108.8	N1—P1—C7	105.68 (9)
C31B—C32B—H32D	109.5	N1—P1—C1	102.94 (9)
C31B—C32B—H32E	109.5	C7—P1—C1	105.08 (10)
H32D—C32B—H32E	109.5	N1—P1—S1	115.77 (7)
C31B—C32B—H32F	109.5	C7—P1—S1	113.74 (8)
H32D—C32B—H32F	109.5	C1—P1—S1	112.50 (8)
H32E—C32B—H32F	109.5	N1—P2—C13	104.68 (9)
C32B—C31B—C28	114.5 (5)	N1—P2—C19	108.30 (9)
C32B—C31B—H31C	108.6	C13—P2—C19	104.31 (10)
C28—C31B—H31C	108.6	N1—P2—S2	113.97 (7)
C32B—C31B—H31D	108.6	C13—P2—S2	113.18 (7)
C28—C31B—H31D	108.6	C19—P2—S2	111.69 (8)
H31C—C31B—H31D	107.6	N2—P3—C39	106.08 (9)
C38—C33—C34	119.26 (19)	N2—P3—C33	103.69 (9)
C38—C33—P3	117.85 (16)	C39—P3—C33	103.83 (10)
C34—C33—P3	122.70 (16)	N2—P3—S3	115.40 (7)
C35—C34—C33	120.0 (2)	C39—P3—S3	113.83 (8)
C35—C34—H34	120	C33—P3—S3	112.84 (8)
C33—C34—H34	120	N2—P4—C45	104.84 (9)
C36—C35—C34	120.4 (2)	N2—P4—C51	107.95 (9)
C36—C35—H35	119.8	C45—P4—C51	104.40 (10)
C34—C35—H35	119.8	N2—P4—S4	114.04 (7)
C35—C36—C37	119.9 (2)	C45—P4—S4	113.23 (7)
C35—C36—H36	120.1	C51—P4—S4	111.68 (8)
			
C6—C1—C2—C3	−0.5 (3)	C59—C60—C61—C62	0.0 (3)
P1—C1—C2—C3	−177.76 (16)	C63—C60—C61—C62	178.6 (2)
C1—C2—C3—C4	−0.1 (3)	C60—C61—C62—C57	−1.2 (3)
C2—C3—C4—C5	0.4 (3)	C58—C57—C62—C61	1.0 (3)
C3—C4—C5—C6	0.0 (3)	N2—C57—C62—C61	178.31 (18)
C4—C5—C6—C1	−0.6 (3)	C61—C60—C63—C64	−177.2 (2)
C2—C1—C6—C5	0.9 (3)	C59—C60—C63—C64	1.3 (3)
P1—C1—C6—C5	177.99 (17)	C26—C25—N1—P1	51.1 (2)
C12—C7—C8—C9	0.7 (3)	C30—C25—N1—P1	−126.23 (18)
P1—C7—C8—C9	178.32 (17)	C26—C25—N1—P2	−120.22 (19)
C7—C8—C9—C10	−1.0 (3)	C30—C25—N1—P2	62.5 (2)
C8—C9—C10—C11	0.3 (4)	C58—C57—N2—P3	50.0 (2)
C9—C10—C11—C12	0.5 (4)	C62—C57—N2—P3	−127.31 (18)
C10—C11—C12—C7	−0.7 (3)	C58—C57—N2—P4	−120.15 (18)
C8—C7—C12—C11	0.1 (3)	C62—C57—N2—P4	62.5 (2)
P1—C7—C12—C11	−177.45 (17)	C25—N1—P1—C7	146.52 (16)
C18—C13—C14—C15	1.6 (3)	P2—N1—P1—C7	−43.21 (16)
P2—C13—C14—C15	176.79 (17)	C25—N1—P1—C1	36.54 (18)
C13—C14—C15—C16	−2.2 (3)	P2—N1—P1—C1	−153.19 (13)
C14—C15—C16—C17	1.0 (3)	C25—N1—P1—S1	−86.61 (16)
C15—C16—C17—C18	0.7 (3)	P2—N1—P1—S1	83.65 (13)
C16—C17—C18—C13	−1.3 (3)	C8—C7—P1—N1	130.08 (17)
C14—C13—C18—C17	0.1 (3)	C12—C7—P1—N1	−52.36 (19)
P2—C13—C18—C17	−175.20 (17)	C8—C7—P1—C1	−121.48 (18)
C24—C19—C20—C21	−1.3 (3)	C12—C7—P1—C1	56.09 (19)
P2—C19—C20—C21	−175.59 (16)	C8—C7—P1—S1	2.00 (19)
C19—C20—C21—C22	0.8 (3)	C12—C7—P1—S1	179.56 (15)
C20—C21—C22—C23	0.4 (3)	C2—C1—P1—N1	−142.13 (17)
C21—C22—C23—C24	−1.0 (3)	C6—C1—P1—N1	40.7 (2)
C22—C23—C24—C19	0.5 (3)	C2—C1—P1—C7	107.43 (18)
C20—C19—C24—C23	0.7 (3)	C6—C1—P1—C7	−69.7 (2)
P2—C19—C24—C23	175.22 (17)	C2—C1—P1—S1	−16.83 (19)
C30—C25—C26—C27	0.4 (3)	C6—C1—P1—S1	166.03 (16)
N1—C25—C26—C27	−176.92 (19)	C25—N1—P2—C13	51.34 (17)
C25—C26—C27—C28	−1.3 (3)	P1—N1—P2—C13	−119.00 (14)
C26—C27—C28—C29	1.1 (3)	C25—N1—P2—C19	162.19 (16)
C26—C27—C28—C31A	−176.5 (3)	P1—N1—P2—C19	−8.16 (17)
C26—C27—C28—C31B	−179.7 (3)	C25—N1—P2—S2	−72.84 (16)
C27—C28—C29—C30	0.0 (3)	P1—N1—P2—S2	116.82 (12)
C31A—C28—C29—C30	176.8 (4)	C14—C13—P2—N1	33.0 (2)
C31B—C28—C29—C30	−179.3 (3)	C18—C13—P2—N1	−151.82 (17)
C28—C29—C30—C25	−0.9 (3)	C14—C13—P2—C19	−80.73 (19)
C26—C25—C30—C29	0.7 (3)	C18—C13—P2—C19	94.49 (18)
N1—C25—C30—C29	178.00 (19)	C14—C13—P2—S2	157.65 (16)
C29—C28—C31A—C32A	−7.0 (6)	C18—C13—P2—S2	−27.13 (19)
C27—C28—C31A—C32A	169.9 (3)	C24—C19—P2—N1	115.33 (17)
C31B—C28—C31A—C32A	−17.6 (8)	C20—C19—P2—N1	−70.27 (19)
C29—C28—C31B—C32B	−94.2 (6)	C24—C19—P2—C13	−133.58 (17)
C27—C28—C31B—C32B	86.6 (7)	C20—C19—P2—C13	40.82 (19)
C31A—C28—C31B—C32B	77.5 (10)	C24—C19—P2—S2	−10.98 (18)
C38—C33—C34—C35	1.0 (3)	C20—C19—P2—S2	163.42 (15)
P3—C33—C34—C35	175.95 (17)	C57—N2—P3—C39	146.55 (16)
C33—C34—C35—C36	−0.6 (3)	P4—N2—P3—C39	−44.39 (15)
C34—C35—C36—C37	−0.2 (3)	C57—N2—P3—C33	37.51 (17)
C35—C36—C37—C38	0.6 (3)	P4—N2—P3—C33	−153.44 (13)
C36—C37—C38—C33	−0.2 (3)	C57—N2—P3—S3	−86.40 (15)
C34—C33—C38—C37	−0.5 (3)	P4—N2—P3—S3	82.65 (13)
P3—C33—C38—C37	−175.77 (16)	C40—C39—P3—N2	130.71 (17)
C44—C39—C40—C41	1.2 (3)	C44—C39—P3—N2	−52.92 (19)
P3—C39—C40—C41	177.63 (17)	C40—C39—P3—C33	−120.34 (17)
C39—C40—C41—C42	−0.9 (3)	C44—C39—P3—C33	56.03 (19)
C40—C41—C42—C43	0.1 (4)	C40—C39—P3—S3	2.73 (19)
C41—C42—C43—C44	0.3 (4)	C44—C39—P3—S3	179.10 (15)
C42—C43—C44—C39	0.1 (3)	C38—C33—P3—N2	−146.72 (16)
C40—C39—C44—C43	−0.8 (3)	C34—C33—P3—N2	38.2 (2)
P3—C39—C44—C43	−177.17 (17)	C38—C33—P3—C39	102.57 (18)
C50—C45—C46—C47	0.8 (3)	C34—C33—P3—C39	−72.49 (19)
P4—C45—C46—C47	175.98 (17)	C38—C33—P3—S3	−21.15 (19)
C45—C46—C47—C48	−1.0 (3)	C34—C33—P3—S3	163.79 (16)
C46—C47—C48—C49	0.3 (3)	C57—N2—P4—C45	52.68 (17)
C47—C48—C49—C50	0.6 (4)	P3—N2—P4—C45	−116.47 (13)
C48—C49—C50—C45	−0.8 (3)	C57—N2—P4—C51	163.55 (15)
C46—C45—C50—C49	0.2 (3)	P3—N2—P4—C51	−5.60 (16)
P4—C45—C50—C49	−175.19 (17)	C57—N2—P4—S4	−71.72 (16)
C56—C51—C52—C53	−1.6 (3)	P3—N2—P4—S4	119.13 (11)
P4—C51—C52—C53	−176.41 (16)	C46—C45—P4—N2	32.5 (2)
C51—C52—C53—C54	1.0 (3)	C50—C45—P4—N2	−152.18 (17)
C52—C53—C54—C55	0.2 (3)	C46—C45—P4—C51	−80.85 (19)
C53—C54—C55—C56	−0.7 (3)	C50—C45—P4—C51	94.42 (18)
C54—C55—C56—C51	0.1 (3)	C46—C45—P4—S4	157.47 (16)
C52—C51—C56—C55	1.0 (3)	C50—C45—P4—S4	−27.3 (2)
P4—C51—C56—C55	176.08 (16)	C52—C51—P4—N2	−73.50 (18)
C62—C57—C58—C59	0.3 (3)	C56—C51—P4—N2	111.58 (17)
N2—C57—C58—C59	−176.99 (18)	C52—C51—P4—C45	37.67 (19)
C57—C58—C59—C60	−1.5 (3)	C56—C51—P4—C45	−137.25 (16)
C58—C59—C60—C61	1.3 (3)	C52—C51—P4—S4	160.37 (15)
C58—C59—C60—C63	−177.3 (2)	C56—C51—P4—S4	−14.55 (18)

### Hydrogen-bond geometry (Å, º)

Cg1 and Cg2 are the centroids of the C19–C24 and C57–C62 rings, respectively.

**Table:**

*D*—H···*A*	*D*—H	H···*A*	*D*···*A*	*D*—H···*A*
C17—H17···*Cg*2^i^	0.95	2.97	3.759 (3)	141
C64—H64*A*···*Cg*1^ii^	0.98	2.88	3.767 (3)	151

Symmetry codes: (i) −*x*+2, −*y*, −*z*+1; (ii) *x*−1, *y*, *z*+1.
